# Role of Mesenchymal Stem Cells and Extracellular Vesicles in Idiopathic Pulmonary Fibrosis

**DOI:** 10.3390/ijms231911212

**Published:** 2022-09-23

**Authors:** Sevindzh Kletukhina, Guzel Mutallapova, Angelina Titova, Marina Gomzikova

**Affiliations:** 1 Laboratory of Intercellular Communication, Kazan Federal University, 420008 Kazan, Russia; 2 Morphology and General Pathology Department, Kazan Federal University, 420008 Kazan, Russia

**Keywords:** mesenchymal stem cells, extracellular vesicles, pulmonary fibrosis, lung damage, mesenchymal stem cells derived extracellular vesicles

## Abstract

Idiopathic pulmonary fibrosis (IPF) is a progressive interstitial fibrotic disease that leads to disability and death within 5 years of diagnosis. Pulmonary fibrosis is a disease with a multifactorial etiology. The concept of aberrant regeneration of the pulmonary epithelium reveals the pathogenesis of IPF, according to which repeated damage and death of alveolar epithelial cells is the main mechanism leading to the development of progressive IPF. Cell death provokes the migration, proliferation and activation of fibroblasts, which overproduce extracellular matrix, resulting in fibrotic deformity of the lung tissue. Mesenchymal stem cells (MSCs) and extracellular vesicles (EVs) are promising therapies for pulmonary fibrosis. MSCs, and EVs derived from MSCs, modulate the activity of immune cells, inhibit the expression of profibrotic genes, reduce collagen deposition and promote the repair of damaged lung tissue. This review considers the molecular mechanisms of the development of IPF and the multifaceted role of MSCs in the therapy of IPF. Currently, EVs-MSCs are regarded as a promising cell-free therapy tool, so in this review we discuss the results available to date of the use of EVs-MSCs for lung tissue repair.

## 1. Introduction

Idiopathic pulmonary fibrosis (IPF) is a specific form of idiopathic interstitial pneumonia. It has been shown to have a high mortality rate and an average of 2 to 3 years lifespan after diagnosis [1]. The disease has been shown to occur in middle or older age people. Primary symptoms of IPF include dyspnoea, hypoxaemia, radiographically marked pulmonary infiltrates and accumulation of fibroblasts in the tissue. These symptoms are as a result of the initial scarring of the lung tissues caused by IPF [2].

IPF has been shown to be caused by damages to alveolar epithelial cells due to genetic, environmental and immunological factors, which leads to metabolic dysfunction, aberrant activation of damaged epithelial cells and pathological epithelial repair. Molecular changes in the alveolar epithelial cells during EMT-induced damage causes the cells to actively express profibrotic factors leading to migration, proliferation, differentiation of fibroblasts and myofibroblasts and subsequent accumulation of extracellular matrix, thereby leading to pulmonary fibrosis [3,4,5]. However, the etiology of this progressive parenchymal disease is still unknown.

There are several factors that contribute to the development of IPF. These include and may not be limited to autoimmune/connective tissue diseases (e.g., rheumatoid arthritis or scleroderma) [6,7,8]; antiarrhythmic drugs administration (amiodarone) [9,10]; chemotherapeutic agents (bleomycin) [11]; antimicrobial agents (nitrofurantoin) [12]; smoking [13]; infectious agents such as mycoplasma [14], legionella [15], rickettsia [16]; viruses (e.g., SARS-CoV-2) [17]; radiation [18,19]; occupational exposures (mining; production of ceramic, acid- and fire-resistant materials; work with sandblasters) [20,21,22]; toxic fumes and gases [23]; genetic predisposition [24,25,26]; diabetes [27]; and gastroesophageal reflux disease [28,29]. The therapeutic modality is determined by the etiology of the fibrosis, e.g., discontinuing the drug that caused the fibrosis, preventing allergic or occupational diseases, or treating the underlying autoimmune disease with immunosuppressive drugs [30].

Current therapies for IPF include lung transplantation, pharmacotherapy and cell therapy. Transplantation has the potential of increasing the quality and life span of patients with lung diseases. However, shortage of donor material, risk of acute graft versus host disease and infection, limit the application of lung transplantation as a therapy measure [31,32]. Pharmacotherapy measures for IPF are predominantly limited with the use of antifibrotic drugs such as pirfenidone and nintedanib. These drugs slow disease progression but do not repair damaged lung tissue and have a number of side effects [33,34,35,36]. Cell therapy for pulmonary fibrosis (PF) involves the use of mesenchymal stem cells (MSCs). MSCs are polypotent cells, capable of differentiating into various cell types and providing immunomodulatory, antiproliferative and anti-inflammatory effects [37,38,39,40]. MSCs secrete growth factors, anti-inflammatory cytokines, chemokines and basic proteins that reduce the deposition of extracellular matrix (ECM) and collagen, as well as promote alveolar repair [41]. However, with MSCs therapy, there is a risk of their differentiation into fibroblasts which produce collagen. Undesirable differentiation and accumulation of mutations during cultivation limit the use of MSCs in clinical practice [42,43,44].

Extracellular vesicles naturally released from MSCs (nEVs-MSCs) are an alternative to cell therapy. It has been shown that the positive effects observed from the use of nEVs- MSCs in IPF therapy has to do with their ability to suppress the activity of growth factors, chemokines and cytokines that stimulate fibrosis development, similarly observed to parental cells [45,46]. It is worth mentioning that injection of nEVs-MSCs, in contrast to MSCs transplantation, does not cause arrhythmia, tumour formation and calcification in the tissues [47,48]. To increase the yield of EVs, protocols are being developed to obtain induced vesicles. These induced vesicles exhibit immunosuppressive properties, similarly observed in natural EVs and parental cells. These immunosuppressive properties may have a positive effect on the therapy of IPF [49,50]. Thus, the use of mesenchymal stem cells extracellular vesicles has reason to be of high interest in the treatment of IPF.

## 2. Fibroblast Heterogeneity in Pulmonary Fibrosis

IPF is the chronic damage of the alveolar epithelium resulting in its inflammation, synthesis and deposition of collagen in the lung interstitium. Chronic inflammation, which is often associated with the formation of a patchy interstitial infiltrate of lymphocytes and plasma cells, has long been thought to be the key mechanism underlying IPF [51,52]. Pulmonary fibrosis was previously thought to be a purely inflammatory process, but, according to current understanding of the pathogenesis of the disease, IPF is primarily characterized by a process of pulmonary fibrosis. Epithelial cell damage and apoptosis represent the initial step in the development of pulmonary fibrosis [53,54]. Various agents, such as allergens, radiation, chemicals, viruses, cigarette smoke, AOFs (active oxygen forms) cause damage to alveolar epithelial cells, which contributes to an inflammatory response [55,56,57,58]. Damaged lung epithelial cells release proinflammatory mediators, namely chemokines, which induce recruitment of circulating blood monocytes, fibrocytes and bone marrow progenitor fibroblast cells [59] to sites of damage [60,61]. Inflammatory cells actively secrete profibrotic activated cytokines and fibrocytes, which subsequently, infiltrate tissue, damage sites of infiltration and promote pulmonary fibrosis by differentiating into active collagen-producing fibroblasts and myofibroblasts [62,63].

Fibrogenesis results from the repeated microdamage of alveolar epithelial cells, followed by the production of factors responsible for activation of a fibrotic program leading to an abbreviated repair of the epithelial cells. This process known as tissue damage-induced epithelial–mesenchymal transition (EMT) on the one hand promotes wound healing and repair of damaged tissue, but on the other hand promotes fibrosis [64,65]. In pathological processes such as fibrosis and in wound healing, the change of epithelial cells to the mesenchymal phenotype is believed to be a necessary process. Epithelial and endothelial cells divide, migrate and participate in the regeneration of damaged tissue [66]. The molecular changes that occur to alveolar epithelial cells during EMT allow them to acquire the mesenchymal cell phenotype. They begin to actively express growth factors (TGF-β, TNF-α, HGF, interleukin-4), secrete numerous mediators (matrix metalloproteinases, angiogenesis inhibitors, and procoagulation factors) leading to their further migration, proliferation, differentiation of fibroblasts into myofibroblasts, and accumulation of extracellular matrix components [67,68,69]. The deposition of excessive amounts of extracellular matrix components leads to an abnormal increase in tissue stiffness. Increased stiffness of lung tissue causes PGE2 (an autocrine inhibitor of fibrogenesis) synthesis suppression, induces further activation of fibroblasts through mechanotransduction pathways and their differentiation into myofibroblasts, thereby contributing to IPF progression [70,71,72,73]. The formed myofibroblasts release numerous profibrotic mediators and synthesize components of the extracellular matrix such as collagen and fibronectin [74,75]. Myofibroblasts, due to their contractile properties, are able to manipulate fibers of the extracellular matrix to heal open wounds, and influence inflammation. All these processes induced by myofibroblasts, in turn, contribute to fibrosis and PF progression.

**Figure 1:**
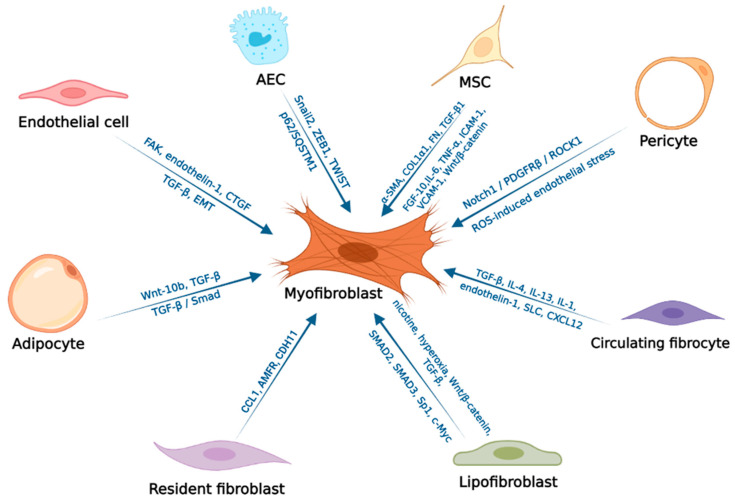
Pathways of myofibroblast formation during IPF. The figure shows the different cell types that respond to stimulation by profibrotic growth factors (TGF-β, CTGF), cytokines (IL-1, IL-13, IL-4, IL-6, TNF-α), signaling pathways (Wnt/β-catenin, Notch1/PDGFRβ/ROCK1), chemokines (CXCL12, SLC, CCL1), specific EMT transcription factors (Snail2, ZEB1, TWIST, p62/SQSTM1), adhesion molecules (ICAM-1,VCAM-1, FAK and CDH-11), hyperoxia, exposure to endothelin-1 and nicotine, overexpression of α-SMA, COL1α1 and fibronectin, upregulation of SMAD2, SMAD3, Sp1 and c-Myc differentiated into myofibroblasts [65,70,76,77,78,79,80,81,82,83,84,85,86,87,88,89,90,91,92,93,94,95,96,97,98,99,100,101,102] (Created with BioRender.com, accessed on 4 July 2022).

Myofibroblasts can arise from different cell types such as epithelial cells, mesenchymal stem cells, pericytes, circulating bone marrow fibrocytes, preadipocytes and adipocytes. These are often collectively referred to as fibroblasts (Figure 1). Fibroblasts is the general term often used to describe these heterogeneous cell populations. Stimulation of TGF-β1 is a known factor to activate fibroblasts and transform them into myofibroblasts, which express α-smooth muscle actin (α-SMA) and secrete cytoskeletal and contractile extracellular matrix proteins (Figure 1). TGF-β binds to type I and type II receptor complexes localized on the cell membrane and stimulates receptor activation leading to phosphorylation of SMAD2/3 in the cytosol. Activated SMAD2/3 additionally bind to SMAD4 and travel to the nucleus where they control the expression of target extracellular matrix proteins by binding to the SMAD binding element in the promoter. The expression of these proteins, as previously mentioned, is a characteristic feature of myofibroblasts [76,77]. Myofibroblasts contribute to lung tissue remodeling through collagen production, profibrotic factors and proteins. In this regard, 25-hydroxycholesterol has been shown to be involved in tissue remodeling in fibrosis. 25-hydroxycholesterol induces NF-κβ p65 transport into the nucleus. Activation of NF-κβ signaling promotes enhanced TGF-β1 release, α-SMA expression, collagen I production and matrix metalloproteinase release [78].

Fibroblast differentiation into the myofibroblast phenotype can also be induced by hypoxia and expression of insulin-like growth factor type II (IGF-II). Hypoxia leads to hypermethylation of the Thy-1 promoter in normal human lung fibroblasts and increases the levels of the profibrotic markers: α-SMA, collagen-1, collagen-3, characteristic of the myofibroblastic phenotype [103]. IGF-II expression is activated during IGF. IGF-II transmits signals through IGF1R/IR, PI3K/AKT/GSK3β and JNK/c-Jun receptors, resulting in the increased activity of ACTA2, TIMP1/4, collagen, fibronectin, TGF-β2/3 and SMAD2/3. Due to the expression of these genes, IGF-II induces myofibroblast formation [104].

According to recent studies, lipofibroblasts play an important role in myofibroblast formation. Lipofibroblasts are resident lung fibroblasts that are located in the thickness of the interalveolar septa near type II alveolocytes and can differentiate into myofibroblasts. This makes them able to contribute to pulmonary fibrosis.

Exposure to nicotine, hyperoxia and Wnt/β-catenin signaling induce differentiation of lipofibroblasts into activated myofibroblasts during IPF formation (Figure 1). 

TGF-β expression also promotes myofibroblast formation from lipofibroblasts by upregulating SMAD2, SMAD3, Sp1 and c-Myc (Figure 1). Conversely, thiazolidinediones, metformin—an anti-diabetic drug, and PPARγ signaling are able to convert myofibroblasts back to lipofibroblasts. The use of PPARγ and its agonists to preserve the lipogenic phenotype and prevent differentiation into the myogenic phenotype in IPF can offer an alternative to existing ineffective drugs (pirfenidone and nintedanib) [76,105]. It has also been found that increased FGF-10 expression in lung tissue during IPF promotes fibrosis resolution through its ability to suppress the activation of profibrotic cytokines, especially TGF-β1, and presumably contributes to the differentiation of activated myofibroblasts into lipofibroblasts [76].

After wound healing, myofibroblasts can follow different pathways: they may die due to apoptosis induced by soluble proapoptotic factors (IL-1β, FGF1 and prostaglandin E2 (PGE2)) [106,107,108]; they may be deactivated and turn into fibroblasts; and they may be aged by the CCN family proteins [109]. This does not occur in IPF, as myofibroblasts in fibrous tissues show high resistance to apoptosis, thus ensuring the preservation of highly active cells at the sites of damage. Resistance to apoptosis is most likely due to the persistent activity of TGF-β1 and myofibroblast-induced deposition of extracellular matrix components, leading to prolonged survival and activity of myofibroblasts during fibrosis development [110,111,112].

## 3. Multifaceted Role of MSCs in Pulmonary Fibrosis

MSCs located in the stromal and perivascular compartments in close proximity to fibroblasts are believed to modulate myofibroblast activity.

There is evidence of the effectiveness of MSCs in the therapy of bleomycin-induced fibrotic lung lesions in animals. Stem cells modulate the activity of B-cells by inhibiting their maturation and recruitment to the sites of lung damage during IPF. Modulation of B-cell activity leads to inhibition of the chronic inflammatory process with subsequent formation of fibrotic lesions [113]. On a model of bleomycin-induced lung damage it was shown that MSCs reduce costimulatory protein expression in dendritic cells and monocyte-derived macrophages, reduce their ability to induce antigen-specific T-cell immune responses, and promote generation of immune cells (Treg) and cytokines (IL-10) with immune regulatory functions [114].

Various deleterious factors induce damage and apoptosis of alveolar epithelial cells, which is commonly accompanied by mitochondrial dysfunction. One of the consequences of mitochondrial dysfunction is an increase in AFC in alveolar epithelial cells (AECs), which contribute to further damage, while there is a persistence of apoptosis-resistant myofibroblasts and excessive deposition of extracellular matrix components [115].

The efficacy of PF treatment with MSCs has been shown to be linked to their ability to deliver mitochondria to the damaged epithelial cells [116], reduce collagen deposition [117], promote tissue repair through the secretion of anti-inflammatory and the anti-fibrotic factors they possess [118]. Mitochondrial delivery reverses the effects of mitochondrial dysfunction and maintains normal mitochondrial homeostasis by delivering healthy mitochondria into damaged epithelial cells [116,119]. Gene expression analysis has shown that MSCs powerfully suppress profibrotic genes induced by bleomycin and inhibit pro-inflammatory transcripts [120]. Human MSCs reduce the levels of TGF-β1 and TNF-α by secreting PGE2 and hepatocyte growth factor (HGF). And TGF-β is known to be a key factor influencing the process of extracellular matrix deposition and myofibroblast differentiation. Other favorable factors secreted by MSCs include PGE2 which has the ability to inhibit TGF-β1-induced proliferation of fibroblasts and collagen production by inducing myofibroblast apoptosis through the increased activity of PTEN (phosphatase and tensin homolog); HGF which reduces the deposition of extracellular matrix in alveolar type II (ATII) cells and induces myofibroblast apoptosis through MMP activation; MMP-9 which promotes myofibroblast death by degrading the FN-CCB domain, and MMP-1 which function as a collagenase to prevent lung tissue thickening [118,121]. FGF-2, secreted by adipose tissue stromal cells, can block further cell differentiation. This has been evidenced by observed decreased gene and protein expression of mesenchymal markers and gene expression of extracellular matrix components. FGF-2 treatment increased the matrix metalloproteinase gene expression and decreased the expression of the metalloproteinase gene TIMP-2 inhibitors in tissues [122,123]. The expression of FGF-10 by lung mesenchymal cells is believed to be involved in the suppression of pulmonary fibrosis. It was shown that FGF-10 expression in lung MSCs inversely correlates with fibrosis progression and TGF-β1 expression in activated myofibroblasts. FGF-10 expression was significantly reduced in stromal cells isolated from bronchoalveolar lavage of patients with IPF, in contrast to MSCs of healthy lungs. This confirms that FGF-10 deficiency can be the cause of disease progression and induction of this factor expression can serve as an effective therapy for IPF [124,125,126].

However, there is alternative evidence for the involvement of MSCs in IPF. In addition to having regenerative activity, MSCs have been shown to contribute to the development and progression of pulmonary fibrosis. Overexpression of α-SMA, COL1α1, fibronectin, TGF-β1, IL-6 and TNF-α in MSCs microenvironment induces MSCs differentiation into myofibroblasts, which in turn promotes collagen deposition and accumulation of extracellular matrix components [24,25,26,27]. Thus, in radiation-induced lung fibrosis, increased expression of cytokines TNF-α, TGF-β1 and adhesion molecules ICAM-1 and VCAM-1 in lung epithelial cells and in damaged lung areas induces bone marrow MSCs migration through the circulatory system to the sites of lung damage and their differentiation into myofibroblasts [127]. A similar effect of microenvironment on the regenerative properties of MSCs was investigated on tissue-resistant lung MSCs, human umbilical cord MSCs, and VEGF-positive MSCs actively used for therapeutic purposes [128,129,130,131]. Moreover, it was shown that bleomycin-induced pulmonary fibrosis induces Wnt10a expression and activation of Shh/Gli signaling cascade resulting in myofibroblast differentiation of lung MSCs. The activation of Shh/Glioblastoma system through Wnt/β-catenin signaling control regulates MSCs transition into the myofibroblast phenotype and enhances pulmonary fibrosis [132].

Excessive activation of PDGFRα signaling leads to fibrosis and vascular calcification. Santini et al. showed that in stem cells actively expressing PDGFRα, there is an upregulation of genes involved in fibrosis, namely, growth factor TGF-β1 and fibroblast activation protein (FAP). Subsequently, MSCs differentiate into myofibroblast-like profibrotic cells, thus contributing to the development of fibrosis [84].

In the manifestation of the profibrotic effects of the MSCs, the stage of fibrosis in which the infusion of stem cells was carried out also plays a role. Yan et al. indicate that infusion of MSCs at a later stage of lung damage enhances IPF. MSCs that were infused at an early stage of fibrosis (after 4 h of fibrosis induction time) were safely engrafted into the lung tissues, whereas MSCs injected at a later stage (at days 60 and 120) were detected as myofibroblasts in the lung interstitial space. This is most likely due to the overexpression of profibrotic cytokines, including TGF-β1, which affect MSC differentiation. These data indicate that MSCs infusion in the late stages of the disease enhances scarring in the damaged tissue rather than having a regenerative effect [131].

Despite the controversial properties, MSCs are currently promising candidates for therapeutic use. For the safe application of MSCs as a therapeutic agent, factors such as optimal dosing, age of the cell donor and the phase of fibrosis at which the stem cells are injected should be considered. Stem cells have maximum beneficial effects when stem cells are administered in the early inflammatory phase of pulmonary fibrosis, whereas a negative effect is observed when they are administered in the late phase of fibrosis. The beneficial effect in the early phase is explained by the fact that MSCs express TGF-β1, which is involved in modulation of immune reactions [39]. At the same time, MSCs serve as a target for TGF-β1, contributing to disease progression at later stages. MSCs of old mice, unlike the stem cells of young mice, divide more slowly in vitro and do not exhibit protective properties [133], hence it is recommended to use MSCs of younger donors in therapy.

## 4. Immunologic Regulation of Pulmonary Fibrosis

Studies on the mechanisms of pulmonary fibrosis have demonstrated the importance of cytokines in pulmonary fibrogenesis in animals and humans. Epithelial and mesenchymal cells, T- and B-lymphocytes, macrophages, neutrophils, eosinophils and platelets are potential sources of cytokines in the lung.

Neutrophils are known to play a key role in the progression of pulmonary fibrosis. They are recruited in the initial phases to exert pro-inflammatory effects. An increased percentage of neutrophils in bronchoalveolar lavage (BAL) fluid has been shown to predict early mortality in individuals with IPF [134]. Infiltration and migration of neutrophils into damaged lung tissue is induced in response to overexpression of CXCL2 on resident alveolar macrophages [135], as well as increased expression of CXCL1 and CXCL2/3, IL-8/CXCL8 [136,137]. In addition, neutrophils produce neutrophil elastase (NE), a neutrophil-specific serine protease, which in turn promotes fibroblast proliferation, myofibroblast differentiation and TGFβ1 activation [138].

Hyperexpression of B-cell antibody genes and focal aggregations of these lymphocytes have been observed in the lungs in IPF [139,140,141,142]. In patients with IPF, B-cell-produced antigen–antibody complexes are present in the lung parenchyma, bloodstream and BAL, which trigger cytotoxic and proinflammatory cascades [143,144,145]. The delivery of B-cells to the foci of inflammation is ensured by the expression of a specific mediator-CXCL13, the sources of which are CD68+ macrophage line cells [146].

Macrophages play a recognized role in wound healing and fibrogenesis through the production and release of chemokines that can engage inflammatory cells and lead to proliferation and activation of myofibroblasts [147,148,149]. In a mouse model of bleomycin-induced pulmonary fibrosis, Baran et al. demonstrated that levels of macrophage colony-stimulating factor, which they showed controls mononuclear phagocyte recruitment and CCL2 production, are elevated in BAL in patients with IPF [150]. Repetitive lung injury in a bleomycin-induced pulmonary fibrosis model showed activation of pulmonary capillary endothelial cells (PCEC) and perivascular macrophages, which in turn inhibits alveolar repair and promotes fibrosis. Suppression of the chemokine receptor CXCR7 expressed on PCEC leads to recruitment of perivascular macrophages expressing VEGFR1 and subsequent stimulation of Wnt/β-catenin-dependent activation of Notch Jagged1 ligand with profibrotic consequences [151]. It has been shown that in addition to initiating the immune response, alveolar macrophages do generate reactive oxygen species (ROS), especially mitochondrial H_2_O_2_, which contributes to the development of fibrosis by increasing the expression of TGF-β1 [152,153]. The data obtained by Larson-Casey et al. demonstrate that alveolar macrophages are a major source of TGF-β1. Similarly, their studies suggest that Akt1-mediated mitophagy promotes alveolar macrophage resistance to apoptosis and is essential for the development of pulmonary fibrosis [154].

A study by Scott et al. showed that increased monocyte counts are associated with an increased risk of adverse outcomes in patients with IPF [155]. Classic monocytes have been shown to infiltrate into the fibrotic lung in the early stages of fibrosis and subsequently mature to profibrotic inflammatory alveolar macrophages [156].

Enhanced monocyte migration into the lungs of patients with IPF occurs in response to increased production of CCL2 (or monocyte chemoattractant protein-1, MCP1) by endothelial cells and increased serum CCL2 concentrations [157,158].

The accumulation of mature dendritic cells (DCs) in large numbers with local maturation potential in ectopic lymphoid follicles has been shown to be induced by resident cells that express chemokines such as CCL19, CXCL12 and CCL2, which ultimately contributes to chronic inflammation in IPF [141,159]. TGF-β is able to modulate the accumulation and phenotypic maturation of CD11c+ pulmonary DCs in a mouse model of pulmonary fibrosis. Thus, the absence of TGF-β leads to a decrease in the percentage of CD11c+CD11b+ cells in the group treated with bleomycin [160].

According to the literature, an increased number of mast cells (MCs) are found in the lungs of patients with IPF [161,162]. In addition, increased levels of tryptase (a trypsin-like enzyme found in mast cell granules and basophils) are found in IPF lung tissue samples. In vitro experiments showed that co-culture of human lung MCs with human lung fibroblasts induced MCs activation and stimulated human lung fibroblasts proliferation, which in turn showed a significantly higher growth rate compared to controls [163]. In addition, it has been shown that lung fibrotic ECM regulates MCs function and that degranulating MCs activate TGF-β1 [164]. Overed-Sayer et al. showed a positive correlation between the number of MCs and foci of fibroblasts in patients with IPF, confirming the link between MCs density and mortality from IPF [165].

The T-cell response is known to contribute to the pathogenesis of idiopathic pulmonary fibrosis [166]. In addition, decreased expression of CD28 on circulating T cells is associated with adverse outcomes in patients with IPF [167]. The regulation of IPF by T cells is carried out through the interaction of Fas–Fas ligand (FasL), cAMP chloride channels or T-cells depletion [168,169,170]. T cells such as Th1/Th2, Th17, Th9 and regulatory T cells are thought to contribute most to the progression of IPF. For example, Th1 secrete antifibrotic factors IFN-γ and IL-12, which inhibit fibroblast proliferation and fibrous tissue formation, while the IL-4 and IL-13 released by Th2 stimulate fibroblast proliferation, collagen production and fibroblast differentiation into myofibroblasts, as well as incline macrophages towards the pro-fibrotic phenotype [171,172,173]. Thus, a Th1/Th2 imbalance has been shown to play an important role in the pathogenesis of pulmonary fibrosis. Th17 cells and IL-17 are found in and around inflammatory infiltrates in patients with IPF, confirming their role in fibrosis development and inflammatory progression [142]. In vitro experiments have shown that IL-17 promotes proliferation of pulmonary fibroblasts, which in turn leads to increased synthesis of type I collagen, TGF-β and IL-6 expression [174]. A key cytokine secreted by Th9 cells is IL-9, which has both antifibrotic and profibrotic effects [175]. However, recent studies have shown that Th9 cells do contribute to pulmonary fibrosis by increasing IL-4-mediated Th2 cell differentiation, and IL-9 neutralisation effectively reduces the degree of pulmonary fibrosis [176]. Regulatory T cells can also have both anti- [177] and profibrotic effects [178,179]. Boveda-Ruiz et al. showed that depletion of CD4+CD25+ regulatory T-cells subpopulation at early stages of BLM-induced lung damage is associated with fibrosis reduction, but this depletion later leads to increased lung fibrosis, meaning that Tregs can be harmful at early stages, but protective at later stages of pulmonary fibrosis in mice [180].

Thus, inflammation may act as an important component in the etiology of IPF, and disease progression is accompanied by innate and adaptive immune responses. Therefore, the immunosuppressive properties of MSCs help to suppress inflammation and promote epithelial tissue repair, which makes them a promising tool for the therapy of IPF.

## 5. Effect of MSCs-Derived EVs on Pulmonary Fibrosis

The therapeutic efficacy of EVs-MSCs has been investigated in preclinical animal models and in many diseases and injuries. Currently, there are more than 300 published reports on the therapeutic properties of exosomes/microvesicles/extracellular vesicles derived from MSCs listed in the PubMed database, covering many categories of diseases.

**Figure 2:**
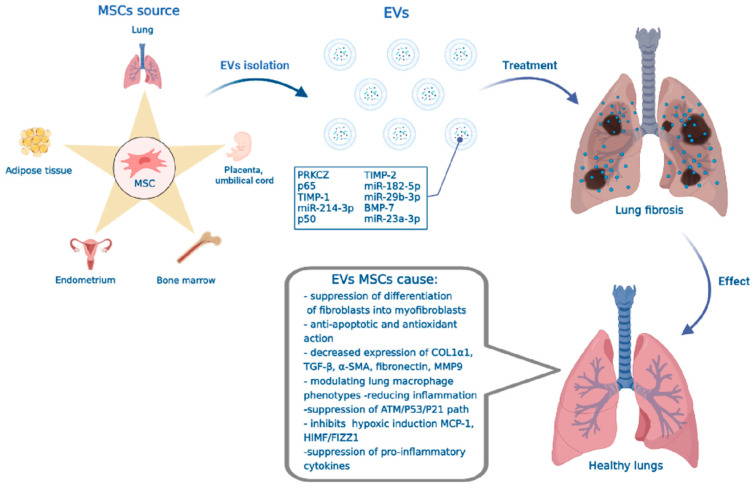
Schematic representation of the lung tissue damage therapy mechanism in IPF by means of mesenchymal stem cell-derived extracellular vesicles. The footnotes in the figure indicate: the composition of EVs-MSCs—proteins (PRKCZ, TIMP-1, TIMP-2, BMP-7), transcription factors (NF-κβ p50, NF-κβ p65), miRNAs (miR-182-5p, miR-214-3p, miR-29b-3p, miR-23a-3p) and therapeutic effect of EVs-MSCs (Created with BioRender.com, accessed on 22 September 2022).

For example, human EVs-MSCs have been shown to be therapeutically effective in an E. coli endotoxin-induced acute lung injury model in mice [181,182]. Intravenous administration of EVs derived from MSCs prevents and treats bleomycin-induced pulmonary fibrosis, with subsequent improvement in both lung morphology and architecture and reduction in collagen deposition. In addition, PF modulates lung macrophage phenotypes by shifting the proportions of pro-inflammatory/classical and non-classical lung monocytes and alveolar macrophages towards the monocyte/macrophage profiles of control mice, thereby reducing inflammation [183]. Xu et al. showed that human umbilical cord EVs-MSCs can inhibit silica gel-induced pulmonary fibrosis, as well as regulate pulmonary function [184]. Lei et al. found that by transferring miR-214-3p in EVs derived from human placental MSCs attenuation of radiation-induced endothelial damage, vascular dysfunction and inflammation as well as pulmonary fibrosis through suppression of the ATM/P53/P21 pathway occurs. They also found that the expression of collagen type 1α-1 (COL1α1), TGF-β, α-SMA, fibronectin and MMP9 genes involved in fibrosis development was significantly reduced after treatment with EVs-MSCs (Figure 2). Moreover, EVs-MSCs increased the expression of several antifibrotic genes including the TIMP-1, TIMP-2 and bone morphogenetic protein-7 (BMP-7) [185]. miR-186, delivered by EVs from bone marrow MSCs (BM-MSCs), reduces fibroblast activation by downregulating the transcription factors SOX4 and DKK1, while slowing the progression of IPF in mice (Figure 2) [186]. Li et al. demonstrated that EVs-MSCs treatment can reduce pulmonary edema, pulmonary dysfunction and inflammation, which were caused by ischemic/reperfusion lung injury in mice [187]. The results of another group of researchers showed that treatment with EVs-MSCs prevents activation of hypoxia signaling, which underlies lung inflammation and the development of pulmonary hypertension in a mouse model. They also showed that administration of EVs-MSCs suppressed hypoxic induction of both MCP-1 and HIMF/FIZZ1 in the lungs, which are strongly activated during lung hypoxia and are potent pro-inflammatory mediators (Figure 2) [188]. EVs BM-MSCs, with miR-29b-3p overexpression, effectively inhibit fibroblast proliferation, invasion migration and differentiation by inhibiting FZD6 expression, contributing to the antifibrotic effects of pulmonary fibroblasts [189]. Treatment of lipopolysaccharide-induced acute lung injury in mice using EVs derived from immature MSCs reduced inflammatory cell accumulation and alveolar septum thickness 48 h after injury compared to the control. In addition, young EVs-MSCs significantly reduced the protein, total cell and neutrophil counts as well as the level of pro-inflammatory cytokine IL-1β and increased the level of anti-inflammatory IL-10 in BAL after 48 h [190]. The results of Sun et al. showed that therapy with EVs from menstrual blood MSCs helped to reduce symptoms of pulmonary fibrosis, reduced collagen deposition and reduced the severity of pulmonary edema. After treatment of bleomycin-induced fibrosis with EVs-MSCs, levels of hydroxyproline (a marker of collagen deposition) and malondialdehyde (a biomarker of oxidative stress in the lungs) were reduced, and levels of glutathione peroxidase (which has an important protective function in the lungs [191]) were increased compared with the untreated group (Figure 2) [192].

Injection of EVs from human umbilical cord MSCs cultured under normal conditions (Nor-EVs) and EVs from human umbilical cord MSCs cultured under hypoxic conditions (Hypo-EVs) significantly reduced total cell count in BAL fluid of eosinophils and proinflammatory mediators (IL-4 and IL-13) in mice with simulated asthma. Compared to Nor-EVs, Hypo-EVs additionally prevented chronic allergic airway remodeling in mice, which was accompanied by a decrease in the expression of the profibrogenic markers α-smooth muscle actin (α-SMA), collagen-1 and TGF-β1-p-smad2/3 signaling pathway [193]. EVs derived from human umbilical cord blood MSCs (hUC-MSCs) therapy reduced inflammation in the lungs and the number of goblet cells, and inhibited lung destruction in a rat model of chronic obstructive pulmonary disease. Additionally, EVs hUC-MSCs partially reduced inflammation through the expression of PRKCZ and p65 and p50 NF-κβ subunits [194]. Treatment of mice with lipopolysaccharide-induced lung injury by intravenous and intratracheal administration of EVs-MSCs significantly reduced inflammatory cell infiltration, septal thickening, lung permeability, total cell and neutrophil counts in BAL. Here, a significant reduction of pro-inflammatory cytokines including interleukin-1β, interleukin-6 and TNF-α in BAL was also shown [195]. Treatment of EVs-MSCs carrying miR-23a-3p and miR-182-5p reversed the progression of lipopolysaccharide-induced lung damage and fibrosis by inhibiting NF-κB and Hedgehog pathways by downregulating Ikbkb and destabilizing IKKβ (Figure 2) [196].

## 6. Clinical Trials of IPF Treatment with MSCs and EVs

A number of clinical trials have been conducted to assess the safety and efficacy of IPF therapy with mesenchymal stem cells. For example, in one study, patients with mild to moderately severe IPF received a single intravenous injection of bone marrow MSCs (20, 100 and 200 × 10^6^/mL). No serious adverse effects were observed, but by 60 weeks after the infusion, there was an average 3.0% decrease in FVC and a 5.4% decrease in carbon monoxide diffusing capacity of the lungs [197]. In another study, patients received intravenous umbilical cord MSCs (10 mL) with a cell density of 5 × 10^6^–1 × 10^7^/mL. There was a slight improvement in lung carbon monoxide diffusion capacity (from 46.3 to 63.1%) and fibrosis score (from 4.6 to 9.8%). Dyspnea scores decreased, with dyspnea at rest decreasing from 3.0 to 1.5; there was a 32.2% increase in maximal inspiratory pressure. The results of the study showed a trend towards an increase in systolic pulmonary artery pressure at 6 and 12 months after administration [198]. In addition, a clinical trial was conducted to investigate the safety of fat-derived MSCs in three endobronchial infusions (0.5 million cells per kg body weight per infusion) in patients with mild to moderate IPF. There were no serious or clinically significant adverse events and no deterioration in functional parameters or quality of life indicators [199].

Another study showed that endobronchial injection of bone marrow MSCs did not cause immediate serious adverse events in patients with IPF, but a certain proportion of patients suffered clinical progression of the disease. Genetic instability of MSCs during cultivation was also observed, which may serve as a limitation for the use of autologous MSCs for IPF therapy. [200]. When intravenous administration of bone marrow MSCs in concentrations of 2 × 10^7^ MSCs/kg (group 1) and 1 × 10^8^ MSCs/kg (group 2), a slower progression of pulmonary fibrosis and a smaller decrease in pulmonary diffusion capacity index were observed in the subjects from group 2 than in the subjects from group 1 [201].

The therapeutic properties of MSCs derived from the placenta were also evaluated. Patients with mild-to-moderate IPF were intravenously injected with MSCs at concentrations of 1 × 10^6^ MSC/kg (*n* = 4) or 2 × 10^6^ MSC/kg (*n* = 4). Further, forced vital capacity (FVC) and pulmonary diffusion capacity were assessed, and a 6 min walk test and chest computed tomography (CT) scan were performed. As a result, there was an increase in FVC (52.5–74.5%) and pulmonary diffusion capacity (29.5–40%); the 6 min walk test and CT scan were unchanged from the baseline [202].

In a small cohort study (*n* = 2) it was demonstrated that nebulisation or intravenous injection of telomerase-positive stem cells increased first-second forced expiratory volume by 14% to 27% and 25% to 70%, respectively [203].

Mesenchymal stem cells have received particular attention because of their ability to modulate the immune system and inhibit inflammation and fibrosis induced by SARS-CoV-2. A study was conducted to assess the safety and efficacy of intravenous administration of Wharton’s jelly-derived MSCs (150 × 10^6^ cells per injection) to patients with COVID-19. The results showed that IL-10 and SDF-1 levels increased after cell therapy, while VEGF, TGF-β, IFN-γ, IL-6 and TNF-α levels decreased. At the same time, the zonal lesion score in both lungs was improved [204].

In 2020, Sengupta et al. conducted the first clinical trial on the safety and efficacy of EVs from allogeneic BM-MSCs (ExoFlo) for therapy of severe COVID-19. Intravenous administration of ExoFlo proved to be safe and was not associated with the occurrence of side effects at any time point. The survival rate was 83%. A single intravenous injection of ExoFlo showed an improvement in oxygenation, a 32% reduction in neutrophil counts and lymphopenia with an increase in mean CD3+, CD4+ and CD8+ lymphocyte counts of approximately 45%. Overall, this study demonstrates that therapy with EVs-MSCs contributed to reversing hypoxia, restoring immunity and suppressing the cytokine storm without treatment-related side effects [205].

## 7. Conclusions

The mechanism of idiopathic pulmonary fibrosis involves a complex interaction of different cell types, factors and signaling pathways. To date, there is a problem with the effectiveness of the applied methods of IPF therapy. Mesenchymal stem cells are the most attractive candidates because they are effective in repairing lung tissue, reducing collagen deposition, suppressing profibrotic genes, inhibiting pro-inflammatory transcripts, and inducing myofibroblast apoptosis. However, the limitations of their use have forced the search for alternative cell-free therapies. EVs-MSCs exhibit the properties of parental cells, promote lung tissue repair and have been confirmed to be safe in clinical trials.

## Data Availability

All data generated or analyzed during this study are included in this published article. The data that support the findings of this study are available from the corresponding author upon request.
